# Validation of a Modified Version of the German Sedentary Behavior Questionnaire

**DOI:** 10.3390/healthcare10050807

**Published:** 2022-04-27

**Authors:** Tobias Kalisch, Christoph Theil, Georg Gosheger, Jan Schwarze, Katharina Voss, Isabell Schoenhals, Burkhard Moellenbeck

**Affiliations:** 1 Department of Orthopedics and Tumor Orthopedics, Muenster University Hospital, Albert-Schweitzer-Campus 1, 48149 Muenster, Germany; christoph.theil@ukmuenster.de (C.T.); georg.gosheger@ukmuenster.de (G.G.); jan.schwarze@ukmuenster.de (J.S.); isabell24@live.de (I.S.); burkhard.moellenbeck@ukmuenster.de (B.M.); 2 Biomechatronics Research Laboratory, Muenster University of Applied Sciences, Buergerkamp 3, 48565 Steinfurt, Germany; katharina.voss@fh-muenster.de

**Keywords:** sedentary behavior, surveillance, questionnaire, validation, accelerometer

## Abstract

*Background*: Physical inactivity and excessive sedentary behavior (SB) are growing public health issues that require surveillance, guidelines, and targeted interventions. In addition to a variety of sophisticated technical methods, questionnaires are still an attractive method for quick, easy, comprehensive, and cost-effective estimation of SB. The aim of this study was to validate a modified version of the widely used Sedentary Behavior Questionnaire (SBQ) compared to waist-worn accelerometers as an objective measurement. Contemporary explanations covering the use of smart devices have been added to the original instrument, and sitting while handwriting was explicated in more detail. *Methods*: Cross-sectional data from an adult sample (*n* = 64, 20–85 y, 25 m, 39 f) were used in this first validation study. Based on prior investigations of the SBQ, analyses were conducted in a gender-specific manner. Criterion validity was assessed using Spearman’s Rho coefficients. The Bland–Altman method was used to test the agreement between self-reported and accelerometer-measured SB time. *Results*: Using the modified SBQ (mSBQ), a significant gender difference in weekly sedentary time was found. Women estimated their sedentary time to be almost 50% higher than men (median 74.5 h vs. 51.0 h). No correlation was found between the questionnaire and accelerometer data for both subgroups (rho ≤ 0.281, *p* ≥ 0.174). Individual differences in daily SB estimation between both methods (in relation to accelerometry) were +3.82 h ± 4.36 h for women and +0.48 h ± 2.58 h for men (*p* < 0.001). *Conclusion*: The modifications to the SBQ did not improve the correlation between self-assessment of SB and objective determination. The reasons for the presented gender-specific overestimation of the participants’ own sedentary time, which contradicts the findings of other studies, remain unclear and need to be investigated further.

## 1. Introduction

Sedentary behavior (SB), including all waking day behaviors in a sitting, reclining or lying posture expending ≤ 1.5 times the resting energy demand, has been identified as an important public health issue [1]. Not least due to the ongoing transformation and digitalization of occupations towards sedentary work, SB is highly prevalent among adults in developed countries [2]. A study in four European countries found that adults aged 20 to 75 years spend an average of nearly nine h/day sitting [3]. Other studies that focused on older adults showed that their average daily sedentary time is even higher, at almost ten hours [4]. Recent research has demonstrated that SB is a highly prevalent, independent chronic disease risk factor. The 2018 Physical Activity Guidelines Advisory Committee (PAGAC) scientific report concluded strong evidence that excessive SB increases the risk for cardiovascular disease, type 2 diabetes, and all-cause mortality [5]. Thereby, the negative health consequences of excessive SB appear to apply even to those who meet the physical activity (PA) recommendations of the World Health Organization (WHO), of at least 150 min of moderate-intensity aerobic activity or 75 min of vigorous-intensity aerobic activity throughout the week [6]. Unfortunately, there is still no international consensus on specific guidelines for sedentary behavior in adults [7].

Accurate measurement of SB is important to facilitate rigorous scientific evaluations of interventions designed to reduce SB. In addition to direct observation, sensor-based techniques such as inclinometers or accelerometers, and various self-report instruments are available. Questionnaires are predominantly used in large-scale studies due to their low administrative costs and their ability to provide additional information about the context of the participants’ behavior [8]. Nevertheless, there are some general limitations, such as a typical underestimation of daily sedentary time, the social desirability of health-promoting behavior, and the inevitable age-related recall bias in self-reports [9,10]. Furthermore, due to its low intensity and habitual nature, SB is difficult to recall [11].

The Sedentary Behavior Questionnaire (SBQ) is one of several specialized multidomain survey instruments designed to capture and quantify SB under everyday conditions. An up-to-date listing and description of established instruments are provided by the Sedentary Behavior Research Network [1]. The SBQ was developed to evaluate the amount of time doing nine context-specific sedentary behaviors on weekdays and weekend days. The English version has first been validated in overweight adults by Rosenberg et al., in 2010 [12]. Only recently, a validation of the English, Spanish, German, and Danish versions of the questionnaire was conducted in older adults [8]. The authors revealed weak correlations between self-reported and objective measures. The average difference between self-reports and accelerometers was −72.9 min/day, with the SBQ underestimating sedentary time. These results are consistent with another European study comparing the Slovenian version of the SBQ with accelerometers in adult sedentary workers. Here, a mean difference of −181 min/day was found between the measurements. Moreover, the data of the two methods were not correlated [13]. To explain the methodological discrepancies, the authors pointed to a ceiling effect in the response options of the SBQ, since exact coverage of a single activity ends at a maximum of 6 h. Furthermore, they mentioned that many sedentary activities resulting from current technological developments are not covered by the SBQ at all [13].

The mentioned findings indicate the need for a revision of the SBQ. In its current form, the SBQ hardly considers contemporary use of technology, especially by mobile devices such as smartphones and tablets. However, there are clear indications that this particular behavior can have negative effects on general physical activity by increasing SB, and, consequently, should be assessed in more detail [14,15,16]. 

For this reason, we modified the SBQ by, first, adding contemporary explanations to the original items that consider the use of smart devices. Second, we restructured specific item content to better capture the activities of ‘sitting while handwriting’ in younger people (in school and professional training) and older people (in everyday living). In this study, we examine the criterion validity of the modified SBQ (mSBQ) to assess SB in adults using waist-worn accelerometers.

## 2. Materials and Methods

### 2.1. Study Design and Participants

In this cross-sectional validation study, we used some data from a previously conducted study that tested the accuracy of accelerometry and inclinometry for the detection of SB in 53 adults. Briefly, we demonstrated that not only inclinometry via thigh-worn devices, but also accelerometry via waist-worn devices can be used to reliably assess SB if appropriate technical settings are used [17]. 

In total, a convenience sample of 64 adults voluntarily participated in this study. The additional 11 subjects included participants who were surveyed about their daily SB and use of smart devices in non-structured interviews during the initial preparation of the mSBQ. All participants gave their written informed consent before participation. Ethical approval was obtained from the local ethics committee (ethics committee of the University of Muenster). The inclusion criteria were age between 20 to 85 years, German language proficiency, and no likely mobility issues (lower limb amputations, endoprostheses, or walking aids). Participants who reported any problems that limited their otherwise normal daily routine in terms of SB and PA were excluded from participation.

### 2.2. Measurement and Procedures

#### 2.2.1. SBQ and mSBQ

The original SBQ version first validated by Rosenberg et al. [12] assessed the amount of time spent on nine context-specific sedentary behaviors on weekdays and weekend days: watching television (1), playing computer/video games (2), listening to music (3), talking on the phone (4), doing paperwork or computer work (5), reading a book or magazine (6), playing a musical instrument (7), doing arts and crafts (8), and driving in a car, bus or riding the train (9). The possible response options were: ‘None’, ‘15 min or less’, ‘30 min’, ‘1 h’, ‘2 h’, ‘3 h’, ‘4 h’, ‘5 h’, or ‘6 h or more’. To obtain daily sedentary time, the data for weekdays and weekend days were summed in the appropriate ratios and averaged [8].

Translation and adaptation from English to German were performed by two independent bilingual translators to identify any discrepancies between the meaning of the translation and the original questionnaire. After final consensus, the following modifications were made: First, item 8 ‘doing artwork or crafts’ was renamed in ‘crafts and hobbies’. Item 7 ‘playing a musical instrument’ was renamed to ‘making music’ and moved to the explanations of item 8. The resulting gap was filled with the new item 7 ‘handwriting’. Second, the explanations for the individual items were revised. For items 1–4 and 6, the use of smart devices (smartphones, tablets, computers, etc.) was considered. For ‘office and computer activities’ (item 5) and ‘handwriting’ (item 7), explicit reference was made to professional and private activities. Do-it-yourself activities were added to the explanations of item 8. The explanations for ‘passenger transportation’ (item 9) remained unchanged and covered car, bus, and train. The opening question (“On a typical weekday/weekend day, how much time do you spend (from when you wake up until you go to bed) doing the following?”) for the nine items was also modified. Now, it explicitly asked about sedentary activities. In the original version of the SBQ, the phrase “sitting while…” only precedes items 3, 4, 6, and 9.

#### 2.2.2. Accelerometry

The ActiGraph wGT3X-BT (ActiGraph LLC, Pensacola, FL, USA) is a small and lightweight triaxial accelerometer that can be attached to various body locations including the waist, wrist, ankle, and thigh. Using proprietary algorithms, the waist-worn monitor can detect body posture (lying, sitting, or standing) and non-wear. The wGT3X-BT monitors were initialized according to the manufacturer’s specifications to record at a frequency of 100 Hz, and the low-frequency extension filter was selected. Data were downloaded from the monitors using the manufacturer’s software, ActiLife v6.13.4. Valid wear times were automatically calculated using the ‘Choi 2011’ wear time algorithm [18]. The sedentary time (i.e., combined sitting/lying time) was calculated based on triaxial accelerometer data (vector magnitude ≤ 150 cpm [19]). The participants were asked to wear the monitors for a minimum of 10 h/day for a period of three to four days, including at least one weekend day. Participants wore the monitor on an elastic belt around the right waist and were instructed to take it off only for water-based activities and sleeping. In addition to the written wearing instructions, participants were provided with a daily log in which they were asked to note any activities (PA or SB) that deviated from their usual behavior during the measurement period. Moreover, reasons for unscheduled discarding of the equipment were requested. For each participant, the following data were calculated: total sedentary time, the average duration of recorded sedentary bouts (i.e., periods with a minimum duration of 10 min and a maximum allowed exceedance of the 150 cpm threshold of 2 min), and the maximum duration of sedentary bouts. The total sedentary time was normalized to an average waking time of 16 h/day (i.e., the typical valid waking wear time determined using a 24-h accelerometer wear protocol in adult subjects [20]).

#### 2.2.3. Statistical Analyses

Before conducting analyses, all variables were examined for normal distribution using the Shapiro–Wilk test. Data were presented as means (M) and standard deviations (SD) (or 95% confidence intervals [CI]), or as medians (Mdn) and 25–75% interquartile ranges (IQR). To improve comparability, the daily SB determined with the mSBQ and the accelerometers were normalized to a 16-h waking period. Paired samples *t*-tests (or Wilcoxon signed-rank tests) were used to assess differences between the means (or medians) of self-reported and monitor-measured daily sedentary time. Internal consistency and inter-item reliability were evaluated using item-total correlations and Cronbach’s coefficient alpha. Criterion validity was assessed using nonparametric Spearman’s rho coefficients between the mSBQ data and accelerometer data. A Bland–Altman plot, including 95% limits of agreement, was used to provide an estimation of the agreement between daily sedentary time measured by the mSBQ and the accelerometer. Correlation analysis was applied to examine associations between the methodological difference in SB detection and other parameters. All statistical analyses were performed using IBM SPSS Statistics 27 (SPSS Inc., Chicago, IL, USA) and the significance level was set at *p* < 0.05.

## 3. Results

Demographic characteristics of the study participants are presented in Table 1. With 25 male participants and 39 female participants, the gender distribution was not balanced. More young (*n* = 28; 20–39 y) and middle-aged (*n* = 25; 40–59 y) adults participated in the study than older adults (*n* = 11, 60–85 y).

All 64 participants provided valid mSBQ data. Based on the results of previous studies utilizing the original SBQ, the data were analyzed on a gender-specific basis and compared between male and female study participants (Table 2). 

Table 3 shows the item-total correlations and Cronbach’s alpha coefficients for the mSBQ. Item-total correlations show how well each item correlated with the composite of the remaining items; correlations ranged from 0.160 to 0.403 and the mean for the item-total correlations was r = 0.227. Only item 3 ‘sitting while listening to music’ exceeded r = 0.30. Chronbach’s alpha of the items ranged from 0.398 to 0.541 and was calculated to be 0.511 for all items, revealing poor internal consistency.

The daily SB data obtained with the mSBQ and accelerometers were normalized to a 16-h waking period. For this purpose, 7 of the 64 mSBQ datasets (10.94%) were limited to a maximal daily sedentary time of 16 h. The original responses of the seven excluded participants (four females, three males) were an average sedentary time of 25.34 ± 10.19 h/day. On average, participants wore the accelerometers for a duration of 2.86 ± 0.75 weekdays and 0.91 ± 0.90 weekend days (total of 3.77 ± 0.58 days). The average daily wear time was 12.80 ± 2.6 h; therefore, the sedentary time was normalized to a waking time of 16 h in 59 of the 64 participants (92.19%). The reasons given by the participants for interruptions in data collection included not putting the accelerometer back on immediately after water-based activities (i), not putting it on until leaving the house in the morning (ii), or taking it off for special activities (swimming, sauna, etc.) (iii). None of the participants reported any behavior (PA or SB) that deviated from their usual routines. The criterion validity of the self-reported daily sedentary time determined with the mSBQ compared to the sedentary time estimated with the accelerometer was calculated for male and female participants (Table 4). No significant correlation was found for both subgroups (*p* ≥ 0.174) and the entire sample (*p* = 0.429).

**Figure 1:**
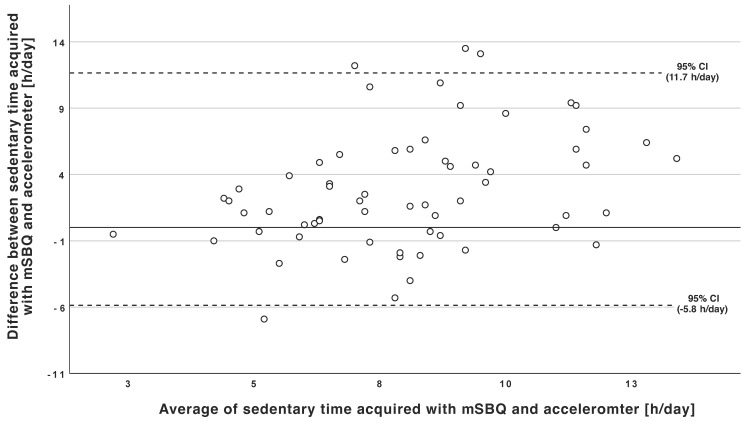
Bland-Altman plot. The solid line shows the mean difference between the sedentary time determined by the mSBQ and accelerometer data; the dashed lines represent the limits of agreement, i.e., the 95% confidence interval (CI).

A Bland–Altman plot was used to graphically compare the differences between accelerometer and self-reported estimations of daily sedentary time (Figure 1). Overall, participants reported higher daily sedentary time using the mSBQ, with a mean difference of +2.91 h/day and a very wide range of limits of agreement (LoA) (upper LoA = 11.67 h/day, lower LoA = −5.84 h/day) compared to the assessment of waist-worn accelerometers.

The outlier-corrected (two of the 64 cases were removed using the boxplot method), methodological differences between the daily sedentary time assessed by the mSBQ and accelerometers were calculated to be 0.48 h ± 2.58 h for male participants, whereas for female participants the average difference was calculated to be 3.82 h ± 4.36 h (*t*-test, *p* < 0.001). No correlation was found between these differences and the participants’ age, BMI, and education level (rho ≤ 0.111; *p* ≥ 0.389), but a significant correlation was observed with their gender (rho = 0.433; *p* < 0.001). With increasing daily total time in sedentary bouts and average length of sedentary bouts the methodological difference decreased (rho ≥ 0.330; *p* ≤ 0.009).

## 4. Discussion

With this study, a modified version of the German SBQ, the mSBQ, was introduced, in which contemporary explanations and minor item restructuring were intended to improve the detection and quantification of SB in adults. Although this is only an initial investigation of the mSBQ and requires further confirmation, the results are remarkable. Instead of underestimating their own SB, as repeatedly reported in studies on self-report methods for measuring SB [9,21], especially with the SBQ [8], participants in the present study massively overestimated their daily sedentary time.

To date, SB questionnaires have mostly been validated in specific populations (e.g., overweight adults [12] or older adults [8]), limiting the generalizability of their properties to general adult populations [11]. Here, we tested the mSBQ on a cohort across the age spectrum. The modifications of the SBQ primarily aimed at improving the acquisition of sedentary time while using smart devices in any age group. It is known, that SB based on screen time is associated with health problems such as the increased risk of obesity, hypertension, hypertriglyceridemia, low bone mineral levels, and psychosocial problems [22]. With technological advances, screen time, including watching television, using a computer or smartphone, is becoming a central component of the daily lives [23] and the most common SB [24]. The SBQ covered screen time in its classical form, but did not consider that watching TV (“streaming”), playing games (“online games”), and reading via mobile smart devices is possible at any time of day (e.g., during transportation, breaks, waiting times, and meals), not just during the typical leisure times in the afternoon or evening [25]. Christensen and coworkers showed that the intensity of smartphone use is almost evenly distributed between 7 a.m. and 10 p.m. [25]. The second modification of the SBQ aimed to capture handwriting, especially among high school and college students. After consultation with younger study participants, this was not adequately captured in the original SBQ by the item “Doing paperwork or computer work (office work, emails, paying bills, etc.)”. However, contrary to our expectations, the modifications of the SBQ did not lead to a reduction of the difference between subjectively and objectively recorded sedentary time—the often-described underestimation of one’s own sedentary time merely turned into an overestimation. Various causes can be discussed for this.

Despite the modification of the SBQ, our version of the test demonstrates the general drawbacks of a paper-and-pencil-based survey method. First, the answers to the individual items are completely independent of each other and only a subsequent data analysis clarifies an exceeding of the maximum possible sedentary time per day. We limited more than 10% of the collected data sets to a total sedentary time of 16 h (i.e., typical wake time) per day, otherwise, the overestimation would have been even more drastic. Here, a technology-supported procedure (i.e., app or online tool) would be much more advantageous, since a plausibility check with feedback can be performed during the test execution. Second, asking for smartphone use, which is often incidental in everyday life, carries the risk of double-counting sedentary activities. For example, the daily train ride to work can be recorded twice with item 9 (‘transportation’) and another smartphone-specific item (e.g., 1, 2, 3, 4, or 6). In fact, SB (e.g., sitting while working and listening to music) is much more likely to be cumulative compared with higher intensity activities [11]. Resolving these conflicts when answering questions about ‘typical behavior’ is likely to be challenging for participants. Nevertheless, in the future, the SBQ should include an instruction to score only one sedentary activity when activities are performed simultaneously. Ultimately, a methodological criticism that has already been repeatedly expressed also applies. When asked about typical behavior, participants are forced to estimate their average behavior, which is very difficult for low-intensity activities like SB [11]. It is known that questionnaires benefit from a short, recent recall frame, which allows remembering specific rather than usual behaviors [26]. In this regard, specific instruments have been developed to assess the SB in the last 7 days (‘SIT-Q-7d’ [11]) or even the previous day (‘PAST’ [27]). In general, estimates of sitting time during TV viewing, nonoccupational computer use, total screen time, and occupational sitting (i.e., behaviors which tend to be more structured and therefore may be more easily recalled) have been repeatedly shown to be more valid than estimates of sitting during transportation, meals, and other sedentary activities [11,28,29]. Furthermore, the quantification of specific sedentary leisure activities, such as household tasks, hobbies, socializing, and listening to music, which typically tend to be performed sporadically and/or for less time, proved to be of lower reliability [11,28,30].

Few studies have examined gender differences in SB assessment using questionnaires. Due to smaller study samples, as well as differences in education level and weight status, the results were difficult to interpret [11,31]. In their English sample, Wijndaele and coworkers showed that reliability was generally stronger among men than women [11]. The authors argued that women may have less structured lives due to the higher prevalence of household and childcare activities, which may compromise the ability to reliably report sedentary time across the day [11]. The methodological differences in SB assessment calculated for men and women in our study point in the same direction but could not be further investigated because detailed information on the participants’ occupational status was not available.

Our results contrast with the most recent findings of other studies that have demonstrated an underestimation of SB by the SBQ. Sansano-Nadal and coworkers showed an underestimation of approximately 1.2 h/day between self-reported and accelerometer measures in 801 English, Spanish, German, and Danish participants when thigh-worn activePAL3c (PAL technologies, Glasgow, Scotland) and Axivity AX3 (AXIVITY Ltd., Newcastle, UK) devices were used [8]. Similar to most other questionnaires [11,27,28,32], the 95% limits of agreement were wide for the assessed daily sedentary time by the mSBQ, making the instrument less suitable for individual-level estimations of sedentary time and for capturing changes in SB in intervention studies.

The underestimation of one’s own time spent sitting, as reported in many studies, has often been explained by certain social desirability, since an inactive lifestyle is nowadays considered undesirable even in old age. A fact that needs to be accounted for in larger cohort studies [33]. During the COVID-19 pandemic, scientific research on general limitations associated with lockdown also brought to the public’s attention the increased risk of developing an inactive lifestyle. These findings, which have been publicly debated for almost two years now, might have changed the ability to self-assess one’s typical sedentary time. To our knowledge, the extent to which the individual perception of pandemic-related changes might affect subjective assessments of PA and SB has not yet been studied. Instead of the known underestimation of SB by social desirability, surveys on SB these days could be influenced in exactly the opposite direction. For working adults, the mandatory relocation to a home office may have resulted in a reduction in PA and an increase in SB that is difficult to self-assess.

This study has some limitations. First, the data were collected only on a small convenience sample, in which the proportion of men and women was different. Second, the objective data on SB were collected using waist-worn accelerometry, although thigh-worn inclinometry has become the gold standard as a criterion measure in recent years [13]. However, based on our own preliminary work [17], we would like to point out that by choosing optimal device settings (i.e., optimized for identifying slow movement), good detection rates can also be achieved using waist-worn accelerometry [17]. Waist-worn accelerometry is still considered the best indicator for reliable determination of wear time, which explains why it is often used in addition to other methods (cf. [8]). Nevertheless, we cannot rule out that some of the methodological differences are due to underreporting of SB by accelerometry. Furthermore, the duration of accelerometric data collection was relatively short and does not allow full conclusions to be drawn about habitual SB. Lastly, we have missed adding a ‘sitting while eating’ item to the mSBQ, which was already objected to in the context of the original SBQ. Sansano-Nadal and coworkers presented the argument that eating breakfast, lunch, and dinner could be one of the reasons for under-reporting SB [8]. However, at least for younger participants, this disadvantage might be less important, as it has been shown that there are correlations between screen time and eating behaviors [34].

## 5. Conclusions

To our knowledge, this is the first modification of the SBQ since its introduction in 2010. Previously, the original SBQ consistently underestimated the total sedentary time and agreed poorly with objective methods, both at the group and individual levels. In contrast, the here-presented mSBQ overestimated the total sedentary time, for women much more than for men. Confirmation of these preliminary results and explanations for the gender differences remain to be carried out on a much larger population. Nevertheless, objective measurement should be the preferred choice when possible.

## Figures and Tables

**Table 1:** Study participant characteristics.

Characteristics	Participants (*n* = 64)
Age (years)	43.5 (IQR: 26.3–57.0)
	range: 20.0–85.0
20–39	43.8%
40–59	39.1%
≥60	17.2%
Sex	25 males, 39 females
Height (cm)	172.6 ± 0.08 range: 156.0–189.0
Weight (kg)	76.2 ± 13.6 range: 53.0–112.0
BMI ^a^	25.5 ± 3.7
	range: 19.0–34.5
Normal and underweight (<25)	29
Overweight (25–29.9)	26
Obese (>30)	9
Education (years)	12.1 (IQR: 10.0–13.0)

Depending on the data distribution, participant characteristics are given either as means with standard deviations or as medians with interquartile ranges (IQR). ^a^ Body mass index (body mass divided by the square of the body height [kg/m^2^]).

**Table 2:** Gender-specific differences in mSBQ items and summary scores.

	Women (*n* = 25)		Men (*n* = 39)			
ST (h/week) per Item	Median	IQR	Median	IQR	Mann- Whitney-U	Significance (*p*)
“TV”	12.0	7.0–16.0	14.0	6.8–16.0	486.5	0.989
2. “Games”	0.0	0.0–2.3	0.5	0.0–7.0	378.5	0.092
3. “Music”	9.0	1.3–22.0	3.3	0.0–7.0	663.0	0.015 *
4. “Phone”	3.5	1.8–11.0	3.0	1.8–5.8	544.0	0.435
5. “Office”	16.0	5.0–31.0	15.5	4.8–29.0	539.0	0.478
6. “Reading”	3.5	2.0–7.0	3.0	0.6–4.5	617.5	0.072
7. “Handwriting”	2.3	0.0–5.0	0.5	0.0–1.5	678.5	0.007 *
8. “Hobbies”	2.5	0.0–11.0	0.0	0.0–6.8	562.0	0.286
9. “Transportation”	6.0	3.5–9.0	6.0	3.5–10.8	470.5	0.814
Accumulated ST (h/week)	74.5	54.8–96.0	51.0	40.3–79.8	641.5	0.034 *
Accumulated ST (h/weekday)	10.5	6.3–13.8	7.0	5.6–12.0	626.0	0.056
Accumulated ST (h/weekend day)	8.0	6.0–13.3	7.0	5.1–10.3	599.5	0.123

ST = sedentary time; * significant result (*p* < 0.05).

**Table 3:** Internal consistency and inter-item reliability of the mSBQ.

Item	Corrected Item- Total Correlation (rho)	Chronbach’s Alpha If Item Deleted (α)
“TV”	0.067	0.501
2. “Games”	0.266	0.541
3. “Music”	0.403	0.473
4. “Phone”	0.273	0.468
5. “Office”	0.169	0.398
6. “Reading”	0.160	0.411
7. “Handwriting”	0.221	0.502
8. “Hobbies”	0.226	0.464
9. “Transportation”	0.261	0.510
All items	0.227	0.511

**Table 4:** Daily average sedentary time assessed by mSBQ and accelerometer.

	mSBQ [h/day]		Accelerometer [h/day]			
Gender	Mean	SD	Mean	SD	rho	*p* Value
Female (*n* = 39)	10.31	±3.65	6.48	±2.42	0.281	0.174
Male (*n* = 25)	8.33	±8.85	6.83	±2.84	0.051	0.758
*t*-test (*p*)	0.048		0.617			
All participants (*n* = 64)	9.53	±3.83	6.62	±2.58	0.101	0.429

SD = Standard deviation; rho = Spearman correlation coefficient.

## Data Availability

The datasets generated and/or analyzed during the current study are not publicly available, but are available from the corresponding author on reasonable request.
